# Lineage-Specific Responses of Tooth Shape in Murine Rodents (Murinae, Rodentia) to Late Miocene Dietary Change in the Siwaliks of Pakistan

**DOI:** 10.1371/journal.pone.0076070

**Published:** 2013-10-14

**Authors:** Yuri Kimura, Louis L. Jacobs, Lawrence J. Flynn

**Affiliations:** 1 Roy M. Huffington Department of Earth Sciences, Southern Methodist University, Dallas, Texas, United States of America; 2 Peabody Museum and Department of Human Evolutionary Biology, Harvard University, Cambridge, Massachusetts, United States of America; University of Birmingham, United Kingdom

## Abstract

Past ecological responses of mammals to climate change are recognized in the fossil record by adaptive significance of morphological variations. To understand the role of dietary behavior on functional adaptations of dental morphology in rodent evolution, we examine evolutionary change of tooth shape in late Miocene Siwalik murine rodents, which experienced a dietary shift toward C_4_ diets during late Miocene ecological change indicated by carbon isotopic evidence. Geometric morphometric analysis in the outline of upper first molars captures dichotomous lineages of Siwalik murines, in agreement with phylogenetic hypotheses of previous studies (two distinct clades: the *Karnimata* and *Progonomys* clades), and indicates lineage-specific functional responses to mechanical properties of their diets. Tooth shapes of the two clades are similar at their sympatric origin but deviate from each other with decreasing overlap through time. Shape change in the *Karnimata* clade is associated with greater efficiency of propalinal chewing for tough diets than in the *Progonomys* clade. Larger body mass in *Karnimata* may be related to exploitation of lower-quality food items, such as grasses, than in smaller-bodied *Progonomys*. The functional and ecophysiological aspects of *Karnimata* exploiting C_4_ grasses are concordant with their isotopic dietary preference relative to *Progonomys*. Lineage-specific selection was differentially greater in *Karnimata*, and a faster rate of shape change toward derived *Karnimata* facilitated inclusion of C_4_ grasses in the diet. Sympatric speciation in these clades is most plausibly explained by interspecific competition on resource utilization between the two, based on comparisons of our results with the carbon isotope data. Interspecific competition with *Karnimata* may have suppressed morphological innovation of the *Progonomys* clade. Pairwise analyses of morphological and carbon isotope data can uncover ecological causes of sympatric speciation and define functional adaptations of teeth to resources.

## Introduction

The influence of climate change on terrestrial mammals can be detected on various temporal and spatial scales, from single-taxon responses such as changes in genetic and phenotypic diversity at the population level to multi-taxon responses such as dispersal and faunal turnover involving immigration and extinction [1]. In modern ecosystems, morphological variation within a species exists along latitudinal and elevational gradients, related to environmental factors such as mean annual temperature and rainfall (e.g., [2], [3]). In the fossil record, temperature change is commonly inferred through oxygen isotope data in deep-sea benthic foraminifera as a proxy of global temperature. The long-term patterns of faunal dynamics and evolutionary diversity in relation to global climate change during the Cenozoic are rigorously studied by sophisticated statistical techniques [4], [5]. Figueirido et al. [5] found by Q-mode factor analysis that increased diversity of successive faunal associations was linked to sustained climatic trends followed by major perturbation events. Although both studies utilized large datasets of North American mammals that span over 65 million years, Alroy et al. [4] challenged the causality of climate to mammal evolution by applying cross-correlation analysis. Over a short time period, however, a cross-correlation series is significant between diversity of small mammals and climate change [6].

Ecological adaptations of mammals have been documented in the context of temporal changes of trophic structure, functional turnover, dietary inference (carbon isotope, microwear, mesowear), and patterns of ecomorphological characters such as body size and height of tooth crown (e.g., [7], [8], [9], [10], [11], [12], [13], [14]). Fossil localities which allow high-resolution time series comparisons between ecological and functional traits of mammals and the local ecosystem are limited, especially for tests of long-term changes. The Neogene fossil record of the Siwalik Group, Pakistan, offers an exceptional opportunity to assess adaptive changes of mammalian communities in response to a dramatic, climatically-controlled vegetation shift in their habitats. More than 50,000 mammal specimens have been collected from Miocene fluvial deposits in the Potwar Plateau, northern Pakistan [7]. In this study, we evaluate the role of dietary behavior on functional adaptations of dental morphology in Siwalik murine rodents from the Potwar Plateau, which experienced a remarkable dietary shift toward greater proportions of C_4_ plants during the C_3_ to C_4_ vegetation shift in the late Miocene [15]. C_4_ plants (most grasses and sedges) have thick lignified walls of bundle sheath cells surrounding nutrient-rich tissues ([16] for review) and have harder phytoliths than those of C_3_ plants ([17], grass vs. squash). Therefore, C_4_ plants are more fibrous, tougher, and less digestible, compared to C_3_ plants (all trees, most shrubs, and some grasses). We analyze tooth shape as the outline of upper first molars by geometric morphometrics of 2D landmarks and test the hypothesis that differential selection pressure acts on dental morphology of murine rodents in different lineages in response to mechanical properties of food items consumed. This study is unique in its fine-scale linkage of dental morphology of individuals with specific diets inferred from carbon isotope composition in molar enamel, as opposed to broad comparisons with global climate or regional vegetation. This approach allows for evaluating the role of functional adaptations of dental morphology in rodent evolution more precisely than in previous studies. In this study, we focus on the timing and direction of morphological change in relation to diet and interspecific competition, rather than rates and mode of morphological evolution.

**Figure 1:**
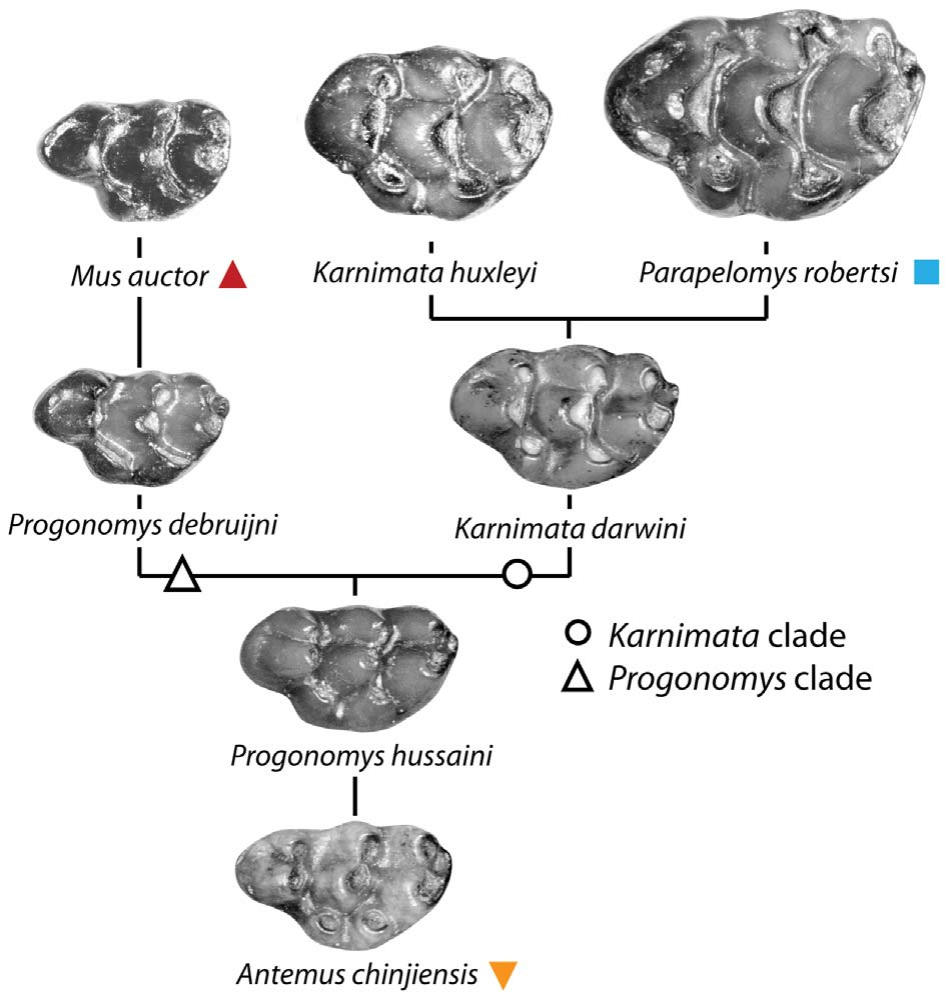
A phylogenetic hypothesis of Siwalik murine rodents proposed by Jacobs [**26**] and Jacobs and Downs [**19**]. Solid symbols correspond to those in Figure 4.

Murine rodents (Old World rats and mice) are the most diverse and abundant of modern mammals and are globally widespread, persistently migrating to new geographical areas [18]. Evidenced by unique dental patterns in molars, this successful rodent group appears to have evolved in South Asia [19], with the oldest definite murine rodent, *Antemus*, appearing as early as 13.8 Ma in the Potwar Plateau [20], [21] and probably older in Sind, Pakistan [22]. *Antemus* is preceded in time by more basal *Potwarmus*, arguably a stem murine [22], [23]. Siwalik murine fossils represent the best and longest record of murine evolution in a chronological framework well-constrained by magnetostratigraphy. After their origin, murines increased in abundance relative to other small mammals at an accelerated rate to dominate over cricetid rodents by ∼11 Ma and successively replace them in ecological niches [24]. However, the diversity of murine species remained low in the Indian subcontinent, between one to four species at single stratigraphic levels [15], until a dramatic increase in the Pliocene [25]. Based on gradual change in dental morphology through the finely-spaced fossil record of Siwalik murines, two fundamental lineages descended from *Antemus* were considered to result primarily from in-situ evolution in northern Pakistan (Figure 1, [19], [26]): the *Progonomys* clade containing *Progonomys* and *Mus* (mice), and the *Karnimata* clade containing *Karnimata*, *Parapelomys*, and possibly *Rattus* (rats). The *Progonomys* clade is different from the *Karnimata* clade in having the anterostyle located more posteriorly [26]. Despite the simplified evolutionary hypothesis of a dichotomy, this interpretation captures overall morphological trends for Siwalik murine rodents [19], [26]. We compare our results of the geometric morphometric analysis with the evolutionary hypothesis of Siwalik murine rodents proposed by Jacobs [26] and Jacobs and Downs [19].

## Materials and Methods

### Sample identification

The upper first molars (M1) of murine fossils, ranging in age from 14.1 to 6.5 Ma in the Siwalik formations of Pakistan, were examined in this study. The advantage of using M1 is that they are systematically the most informative teeth in the Murinae [27]. Temporal changes of dental morphology, if present, appear more clearly in M1 than in any other tooth position. M1 specimens were recovered from the Potwar Plateau by screen-washing in the 1970's to 2000. Among the fossils analyzed in this study, six species of four genera were described based on specimens from localities YGSP 491 (13.8 Ma), YGSP 41 and 430 (13.6 Ma), YGSP 182 (9.2 Ma), and DP 13 (6.5 Ma) [26], [28]. Based on morphology and size of M1, Jacobs and Flynn [21] reported a list of murine fossils from other localities mostly at the generic level. Here, we generally follow Jacobs and Flynn [21] for names of taxa, including ambiguous groups such as “near *Progonomys*”.

**Figure 2:**
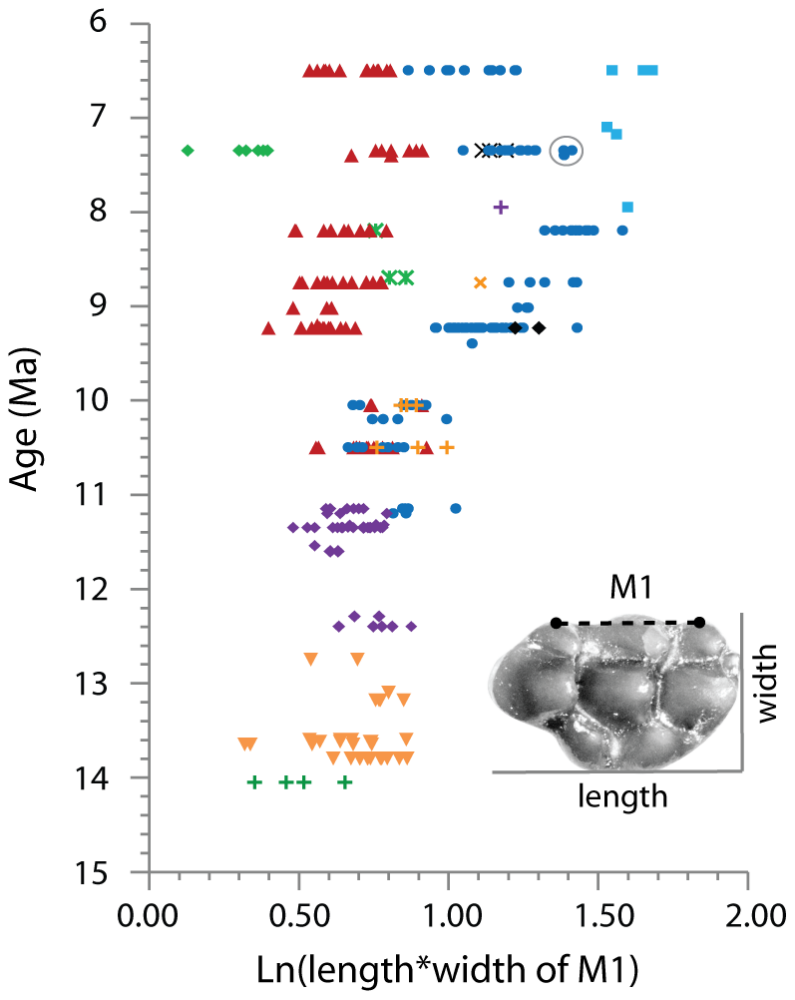
Natural logarithm of length*width on M1 of Siwalik murine rodents. Length was measured on the longitudinal axis of the tooth, which is parallel to the dotted line connecting two reference points on the labial side of the tooth, as in [53]. Symbols: 14.1 Ma, green cross for “near *Antemus*”; 13.8 to 12.8 Ma, inverted orange triangle for *Antemus chinjiensis*; 12.4 Ma, purple diamond for “near *Progonomys*”; 12.3 Ma, purple diamond for *Progonomys* sp.; 11.6 to 11.2 Ma, blue circle for ?*Karnimata*, purple diamond for *Progonomys hussaini*; 10.5 to 10.1 Ma, blue circle for *Karnimata* sp., red triangle for *Progonomys* sp.; 10.5 Ma, orange cross for morphotype 1; 10.1 Ma, orange cross for morphotype 2; 9.4 to 9.0 Ma, black diamond for *Parapodemus* sp., blue circle for *K. darwini* red triangle for *P. debruijni*; 8.8 to 8.2 Ma, blue circle for *Karnimata* sp. (+large *Karnimata* sp.), red triangle for *Progonomys* sp., orange x for morphotype 4, green asterisk for morphotype 7; 8.0 to 7.1 Ma, light blue square for *Parapelomys* sp., blue circle for *Karnimata* sp., purple cross for morphotype 8, black x for morphotype 4, red triangle for *Progonomys* sp. green diamond for *Mus* sp.; 6.5 Ma, light blue square for *Parapelomys robertsi*, blue circle for *K. huxleyi*, red triangle for *M. auctor*. Three samples of *Karnimata* sp. at 7.4 Ma which were grouped with *Karnimata* sp. at 8.2 Ma are circled.

We grouped tooth samples from 36 localities based on age, size, and morphology of M1, so that individuals in the same assemblage show high similarity (see Table S1 of Kimura et al. [15] for the classification). Specimens in single assemblages are hypothesized to represent populations, which could be used to define species given further research. However, separate assemblages of different ages do not necessarily mean different species (e.g., *Progonomys* sp. from 8.7 Ma and *Progonomys* sp. from 8.2 Ma). Specimens used in this study are plotted in Figure 2 (Dataset S1) for tooth size, which is calculated as the natural logarithm of tooth area (length*width). Three specimens (YGSP 52930, 52989, and 27471) of *Karnimata* sp. recovered from 7.4 Ma were grouped with *Karnimata* sp. at 8.2 Ma, rather than with that of 7.4 Ma, because the size of the three specimens matches the range of *Karnimata* sp. at 8.2 Ma. Specimens of *Progonomys* sp. (n = 2 by M1) from YGSP 634 (12.3 Ma) were combined with *P. hussaini*, and *Progonomys* sp. (n = 3 by M1) from YGSP 311 (10.1 Ma) was combined with *Progonomys* sp. at 10.5 Ma due to small sample sizes. Large *Karnimata* sp. (n = 1 by M1) from YGSP 182 (9.2 Ma) was combined with *K. darwini*. In Kimura et al. [15], large *Karnimata* sp. was distinguished from *Karnimata* sp. at 8.2 and 8.8 Ma based on characters of the lower first molars, the presence of a distinct medial anteroconid and the lack of the x-shaped intersection formed by lingual and labial anteroconids, protoconid, and metaconid. However, we could not recognize the two species based on the dental morphology of M1. Tooth terminology follows Jacobs [26] (Figure S1).

All specimens used in this study are housed in the Peabody Museum of Archaeology and Ethnology, Harvard University, and are on long-term loan from the Geological Survey of Pakistan, Islamabad, Pakistan. Museum IDs are given in Dataset S1. No additional permits were required for the described study, which complied with all relevant regulations.

### Geometric morphometrics of tooth outline

The M1 outline of murines is strongly influenced by the relative position of the main cusps except for the protocone, located in the center of the middle chevron, and has been used in geometric morphometric and Fourier analyses (e.g., [29], [30], [31], [32], [33], [34]). A landmark-based geometric morphometric approach was adopted in this study.

**Figure 3:**
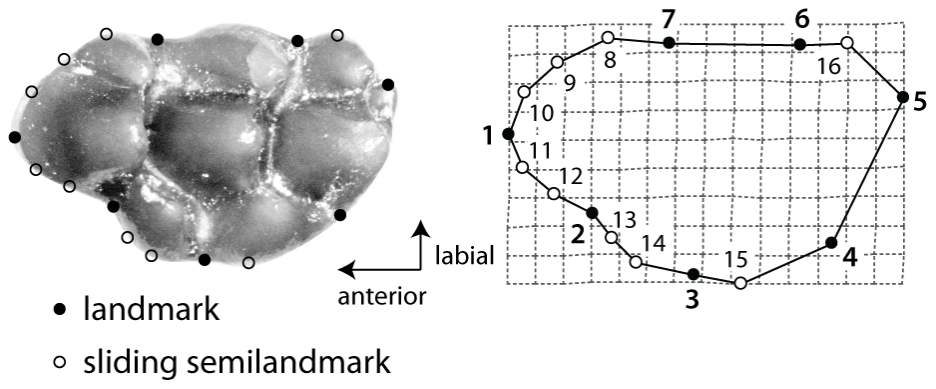
Positions of seven landmarks (solid dots) and nine sliding semilandmarks (open dots) along M1 outline. The specimen is *Karnimata darwini* (YGSP 7692, reversed) in wear stage I.

We selected complete specimens in wear stages I to IV of Lazzari et al. [35], which are lightly to moderately worn teeth (Figure S2). A total of 296 specimens were digitized photographically using a Keyence VHX-1000 digital microscope at Southern Methodist University. The x- and y-coordinates of seven landmarks were obtained along the outline (Figure 3) using tpsDig version 2.16 [36]. These landmarks are points on the local maximum curvature of the outline associated with cusps and valleys between cusps and are equivalent to the Type II landmarks of Bookstein [37]. Nine sliding semilandmarks were placed between the fixed landmarks in the anterior part of the tooth (Figure 3). Sliding semilandmarks are useful to capture the geometry of shape on which definite landmarks are difficult to place, because they are allowed to slide along an outline curve to minimize the amount of shape change between a selected specimen configuration and the average configuration of all specimens [38]. A minimum bending energy criterion was adopted in this study as in Macholán [29], which conducted a geometric morphometric analysis on M1 outline in modern species of *Mus*. In this criterion, a sliding semilandmark point is slid along a tangent line to a curve at the original position in order to find a position that minimizes the bending energy required to deform the average configuration into a specimen configuration, and the adjusted point is projected back to the outline of the specimen configuration ([39] for summary). We checked that PCA scores, including all, of the shape data under the criterion are significantly correlated with those under the minimum Procrustes distance criterion by Procrustes superimposition of the two datasets in the ‘vegan’ package in R [40] (r = 0.98, *p* = 0.0001 by 9999 permutations). In contrast to Macholán [29], we did not place sliding semilandmarks in the posterior part of the tooth, but used one landmark (landmark 4) at the boundary between the base of the enterostyle and hypocone. We reduced the amount of (semi)landmarks on the posterior outline of the tooth because the shape of the posterior outline depends on wear stage due to posterior inclination of cusps in murine rodents. Other minor differences from Macholán [29] are that sliding semilandmarks are substituted for landmarks (8, 14, 15, 16), and that no landmark is placed on the curvature of the paracone.

All landmark configurations were scaled to unit centroid size and superimposed to minimize the sum of squared differences in corresponding landmarks between each specimen and average configuration by the generalized Procrustes analysis of Rohlf and Slice [41]. The superimposed shapes were orthogonally projected to the tangent plane, on which the average configuration is a tangent point to shape space [37], [42], [43]. Partial warp scores (non-uniform shape variables) and a uniform shape component were calculated using α = 0 in the equation for the weight matrix [42]. These procedures were conducted in tpsRelw version 1.49 [38]. Statistical analyses were performed using the shape data that are composed of partial warp scores and uniform shape component scores.

### Euclidean distances and overlap area

To assess patterns of shape change in Siwalik murines, principal component analyses (PCA) were carried out on the covariance matrix of the shape data in PAST [44]. A covariance matrix rather than a correlation matrix was used because the special scaling of partial warps must be retained [45], [46]. The Euclidean distances among specimens are preserved in principal component (PC) axes [47]. In the multidimensional morphospace on PC axes, the distance from basal morphology can be interpreted as the amount of evolutionary change needed to reach a derived shape [48]. The multidimensional distances from the mean of *Antemus chinjiensis* at 13.8 Ma to that of each species were calculated to evaluate the amount of shape change from the basal murine species. We chose to use it over the distance between two successive species normalized by time because the latter always gives a positive value even if shape temporarily fluctuates to basal conditions. In contrast, the distance from the basal species is expected to be positive prior to calculation based on the results of PCA. Ten principal components that explain more than 95% of total variance were subject to the distance calculation. The multidimensional distance between means of two coexisting species was also calculated in R2.15.1 [49]. The 95% bootstrap confidence intervals were computed in the bias-corrected and accelerated method with 9999 randomizations in the ‘boot’ package in R [50]. The confidence intervals between two species were compared simply by the ‘rule of eye’ of Cumming and Finch [51] that *p*≤0.05 when 95% confidence intervals of the two independent groups overlap less than half the average of the half-width of the confidence interval.

Even with the same distance between means, two coexisting species with large intraspecific variation have a greater proportion of morphological overlap in the morphospace than those with small intraspecific variation. Thus, the proportion of morphological overlap area on PC1 and PC2 was measured between coexisting species in ImageJ [52]. A 55% concentration ellipse was utilized because it is less sensitive to outliers than a convex hull.

### Morphometric distance of dental characters

We measured morphometric distances for three dental features, which are related to informative characters to differentiate the *Karnimata* clades from the *Progonomys* clade, using a Keyence VHX-1000 digital microscope. First, the shape of the anterostyle was measured at the base of the cusp by taking the ratio of the minor axis to the major axis of the ellipse-shaped cusp. Specimens were oriented with the anterostyle perpendicular to the microscope lens. Second, the angle of the anterostyle was measured by the acute angle between a longitudinal axis of the tooth and the major axis of the anterostyle. Third, the angle of the enterostyle was measured by the acute angle between the longitudinal axis of the tooth and a line passing the centers of protocone and enterostyle. For the second and third characters, digital photographs taken for the geometric morphometric analysis were utilized. 285 specimens were measured in total. All dental measurements were taken to 0.01 mm accuracy in Keyence VHX-1000 communication software. The longitudinal axis of the tooth is parallel to the reference line of Martín Suárez and Freudenthal [53] that passes through landmarks 8 and 16 of this study.

### Statistical analysis

To examine patterns of covariation between tooth shape and van Dam's [54] index, a two-block partial least-square (PLS) analysis was performed in MorphoJ [55]. The van Dam's [54] index (VD index hereafter) is an ecomorphological measure of grazing diet in Murinae [54]. It reflects space between chevrons on M1 by calculating the ratio of tooth width to the anteroposterior distance between the lingual anterocone and protocone (see [15]). Teeth with a greater VD index have narrower space between the two cusps, which is a better adaptation to grazing diets [54].

The PLS analysis constructs principal components of covariation between the two blocks of variables by using singular value decomposition on the matrix of correlations between the blocks [45], [46], [55]. Permutation tests with 9999 randomizations were used for testing the null hypothesis of complete independence between the blocks. We also obtained the RV coefficient, which is a multivariate analogue of the squared correlation [55], in MorphoJ. The PLS analysis was conducted for all taxa included and for the two clades separately. In the two clades, we separately analyzed two anagenetic lineages, *Karnimata* of the *Karnimata* clade and the *Progonomys* clade without *Mus* sp. at 7.4 Ma. That 7.4 Ma species of *Mus* (green diamonds in figures) was excluded because it may be an immigrant from another region [15]. To avoid confusion, the *Progonomys* clade without *Mus* sp. at 7.4 Ma is expressed as the “*Progonomys* clade” hereafter.

To test for the relationship between tooth shape and size, shape data were regressed on the natural logarithm of centroid size, and multivariate test statistics were computed in tpsRegr version 1.38 [56]. Wilk's lambda was used as a test statistic for the multivariate analysis. The PLS analysis was also conducted to test for covariation between the two sets of variables as well as the RV coefficient.

### Comparison with isotopic dietary inferences

We descriptively compared the temporal changes of tooth shape with paleodietary data [15] inferred from carbon isotope analysis of lower first molar (m1) tooth enamel of the murine species. Cross-correlation is a useful method for finding correlations between two time-series data sets such as mammalian taxa and oxygen isotope data (e.g., [4], [6]). We preferred the side-by-side comparison for tooth shape and carbon isotope data because carbon isotope ratios in teeth are short-term variables, coming from two end-member isotopic sources of vegetation, rather than the long-term variation of tooth shape.

## Results

### Tooth outlines

**Figure 4:**
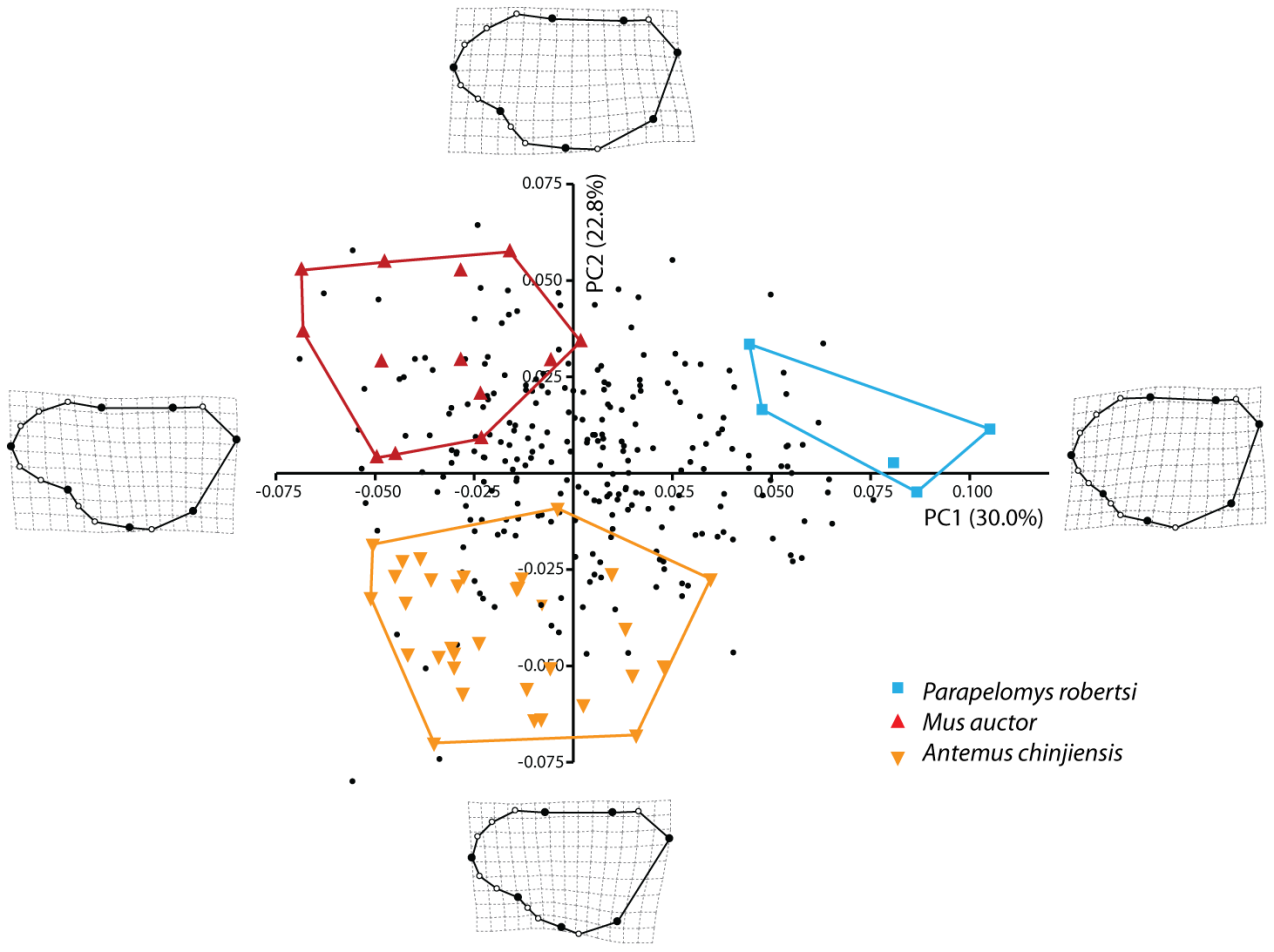
A scatterplot of the first and second PC axes from principal component analysis (PCA) of the shape data in Siwalik murines. All individuals on Dataset S1 were plotted.

**Figure 5:**
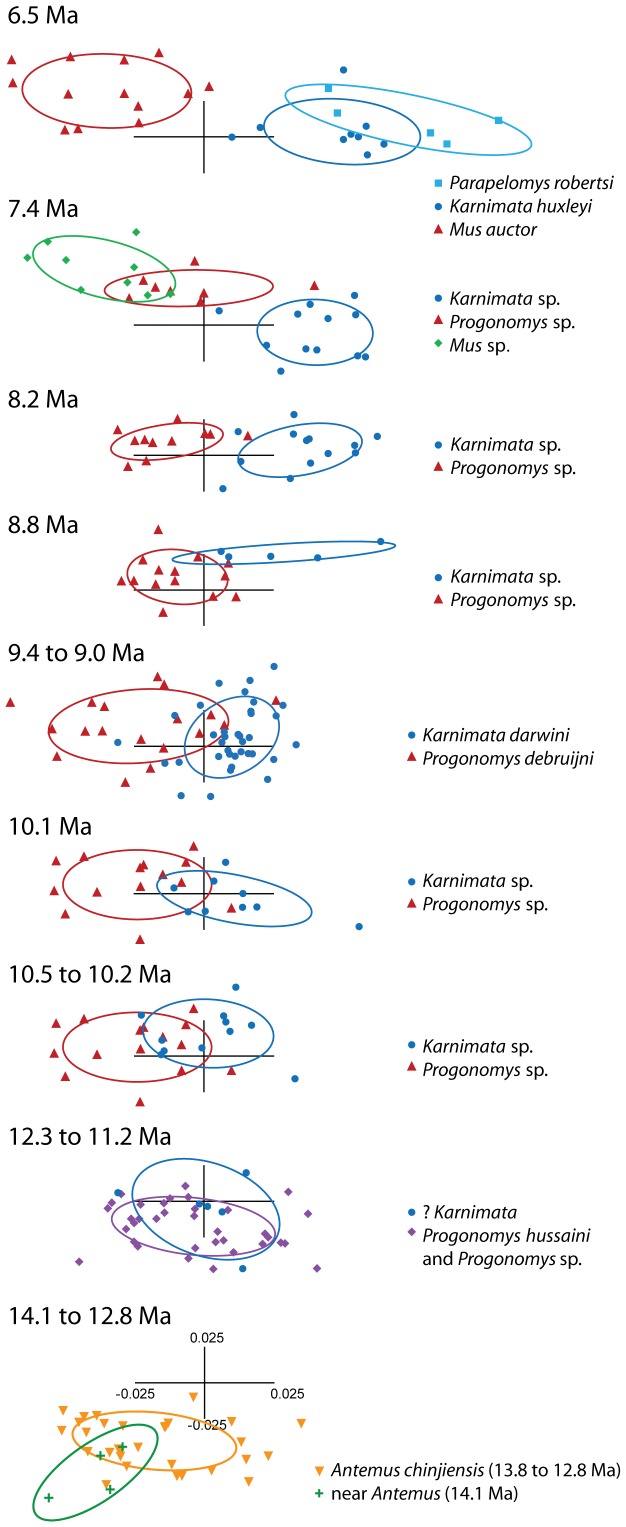
Scatterplots of the first and second PC axes from PCA of **Figure 4** **for major species (n≥5 by M1), showing shape change and overlap area through time.** Symbols as in Figure 2. The ellipses show 55% concentration ellipses. Note that pooled samples of *Progonomys* sp. at 10.5 and 10.1 Ma are repeated.

All individuals of Siwalik murines used in this study fall into a confined morphospace with large overlap among species of the same clade and some overlap between species of similar ages (Figures 4 and 5). PC1 and PC2 account for 30.0% and 22.8% of total variance in tooth shape, respectively (Table S1). On PC1 and PC2, the morphospace is nearly a triangular shape (Figure 4), the apices of which are occupied by the most basal and earliest species, *Antemus chinjiensis*, and two species from the youngest age analyzed here, *Mus auctor* and *Parapelomys robertsi*. Generally, species appearing in younger ages are located further away from *A. chinjiensis*, and divergence between coexisting species increases through time (Figure 5). Despite the small sample size, the mean shape of “near *Antemus*”, which occurred prior to the appearance of *A. chinjiensis*, is located outside that of *A. chinjiensis* (Figure 5).

On the PC1 and PC2, the morphological trajectories toward tooth shape of *Mus auctor* on one side and that of *Parapelomys robertsi* on the other side, both derived from *A. chinjiensis* [19], [26], can be summarized as follows. First, a set of landmarks (2, 13, 14) along the anterior outline of the anterostyle shifts anteriorly to increase the size of the anterostyle; second, landmark 3 along the lingual outline of the enterostyle slightly shifts anterolingually, whereas landmark 4 shifts posterolabially to increase the size of the enterostyle. Leading toward *M. auctor*, a set of landmarks (10, 1, 11, 12) shifts anteriorly to elongate the anterior side of the lingual anterocone, whereas landmarks 2 and 14 along the anterior outline of the anterostyle slightly shift labially and posteriorly, respectively. The three sets of landmarks displace the anterostyle more posteriorly relative to the anterior part of the tooth outline. Landmark 7 shifts lingually; a set of landmarks (6, 16, 5) shifts anterolingually; and a set of landmarks (3, 15, 4) shifts posterolabially. These sets of landmarks make the tooth outline more rectangular. Leading to *P. robertsi*, the set of landmarks (2, 13, 14) shifts anterolingually, with the anterostyle located more anteriorly, whereas the set of landmarks (8, 9, 10, 1, 11) on the anterior side of the tooth shifts posteriorly. A set of landmarks (7, 6) shifts labially, and a set of landmarks (16, 5) shifts anterolabially. The four sets of landmarks make the tooth outline more square.

### Overlap area and Euclidean distance among coexisting species

**Figure 6:**
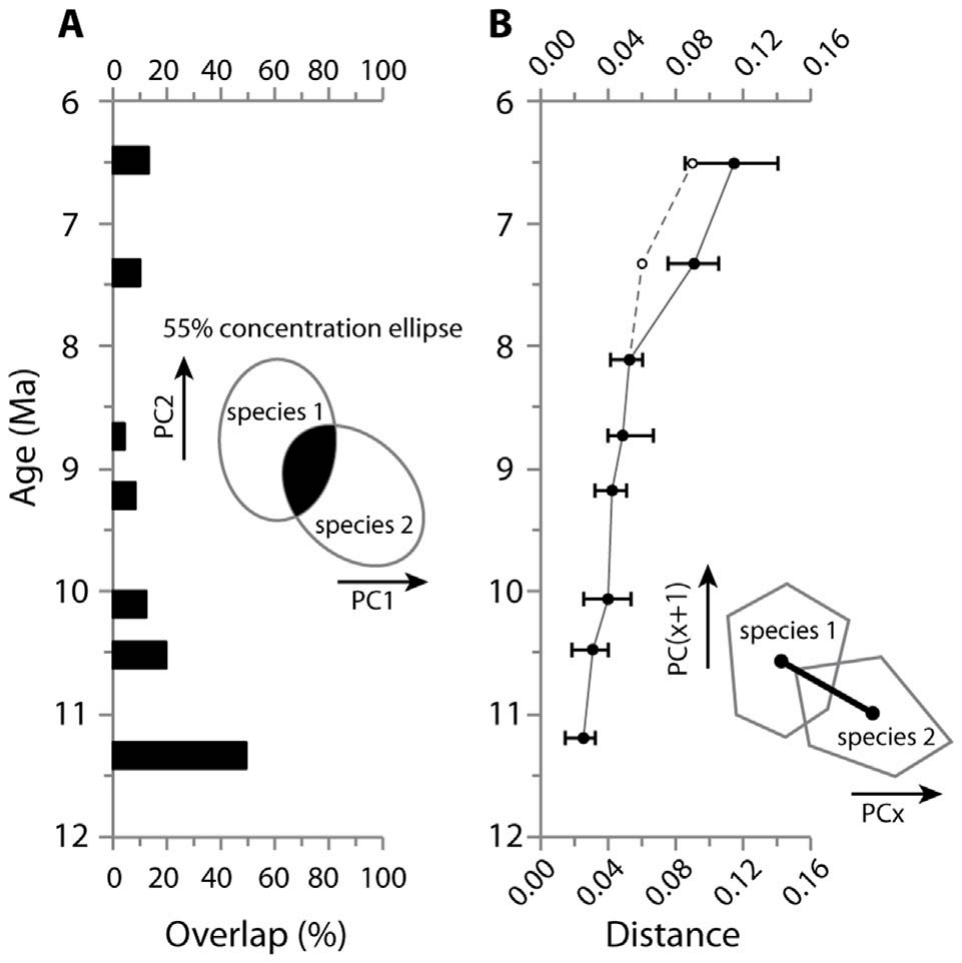
Temporal change of overlap area and Euclidean distances in coexisting species of Siwalik murines. (A) Overlap area on the first and second PC axes among coexisting species. (B) Euclidean distances on 10 principal components. Error bars indicate 95% bootstrap confidence intervals. At 7.4 and 6.5 Ma, where three species were analyzed, the largest distances are shown by solid circle, and distances between *Karnimata* (blue circles in Figure 2) and the “*Progonomys* clade” (red triangles in Figure 2) are shown by open circle. As in Figure 5, pooled samples of *Progonomys* sp. at 10.5 and 10.1 Ma were repeated.

As mentioned above, coexisting species share some proportions of their occupancy in the morphospace, but the morphological overlap of coexisting species decreases through time. Figures 5 and 6 show change in the proportion of the overlap area on PC1 and PC2 shared by coexisting species through time. At the appearance of the two morphotypes, ?*Karnimata* (11.2 Ma) and *Progonomys hussaini* (12.3 to 11.2 Ma) share 49% overlap area (Table S2). By 10.5 to 10.2 Ma, the overlap area shared by *Karnimata* (10.5 to 10.2 Ma) and *Progonomys* (10.5 to 10.1 Ma) decreases to 20%, which is less than half of the overlap area in the previous age. The striking decrease of overlap area may arise in part from the small sample size of ?*Karnimata* (n = 6) relative to *P. hussaini* (n = 35). By 8.2 Ma, *Karnimata* sp. and *Progonomys* sp. do not overlap in the morphospace. When three major species (n≥5 by M1) are present at 7.4 Ma, *Karnimata* sp. does not overlap with the two other species, but *Progonomys* sp. and *Mus* sp. share 10% of the total area occupied by the three species. At 6.5 Ma, the proportion of the overlap area remains similar. Tooth shape of *Mus auctor* is distinct from the two other species, but *P. robertsi* and *K. huxleyi* overlap in shape. There is no overlap between *Karnimata* and the “*Progonomys* clade” (i.e., the *Progonomys* clade without *Mus* sp. at 7.4 Ma) at either 6.5 or 7.4 Ma.

The Euclidean distances between means of two coexisting species were calculated on 10 PCs, which account for 95% of total variance (Figure 6, Table S2). The distance between two coexisting species increases gradually until 8.2 Ma. At 7.4 Ma and 6.5 Ma, when three major species were present, the largest distance was observed between the largest and smallest species (solid dots in Figure 6). The Euclidean distance became significantly large by 7.4 Ma and continues to increase until 6.5 Ma. In *Karnimata* and the “*Progonomys* clade”, the distance significantly increased between 7.4 and 6.5 Ma (open dots in Figure 6).

### Euclidean distance from *A. chinjiensis*

**Figure 7:**
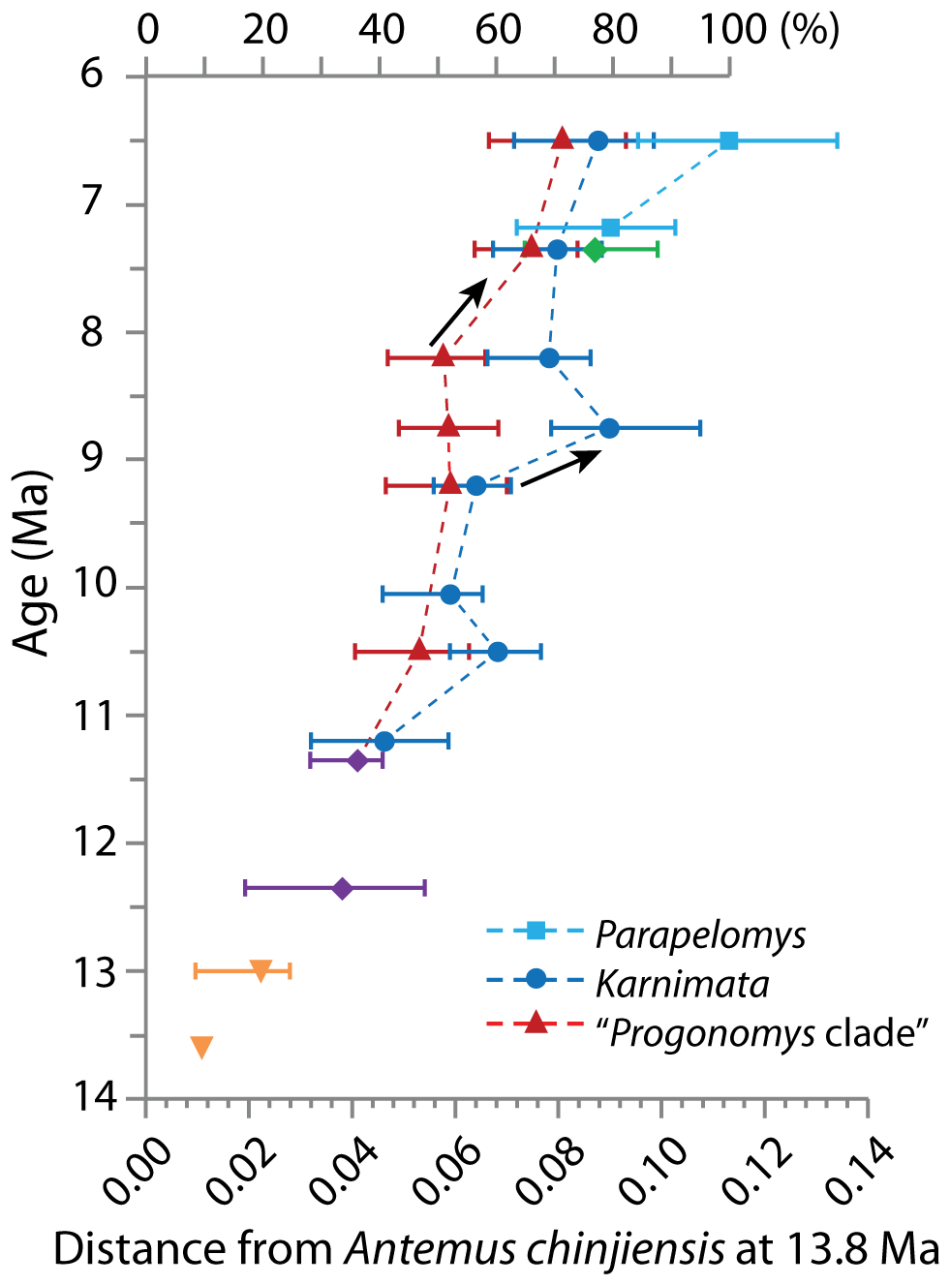
Euclidean distances from *Antemus chinjiensis* at 13.8 **Ma to each species, using 10 principal components.** The percentage on the top of the ordinate axis is expressed as a percentage of morphological change as *A. chinjiensis* at 13.8 Ma is set to be 0%, and *P. robertsi* is set to be 100%. Symbols as in Figure 2. Combined ages: 13.7 to 13.6 Ma for *A. chinjiensis* at 13.6 Ma, 13.2 to 12.8 Ma for *A. chinjiensis* at 13.0 Ma, 11.6 to 11.2 Ma for *P. hussaini*, 10.5 to 10.1 Ma for *Progonomys* sp. at 10.5 Ma, 9.4 to 9.0 Ma for *K. darwini*, 9.2 to 9.0 Ma for *P. debruijni*, 8.0 to 7.1 Ma for *Parapelomys* sp. at 7.2 Ma.

Figure 7 shows the Euclidean distance between the mean of *Antemus chinjiensis* at 13.8 Ma and that of each species (Table S3). Note that Figure 7 displays the amount of deviation from *A. chinjiensis* and thus the difference between two coexisting species on the figure is not equivalent to the Euclidean distances between the two species in Figure 6. On the ordinate axis (top), the amount of deviation from *A. chinjiensis* is also expressed by a percentage of morphological change with *P. robertsi* set at 100%. Dotted lines connecting data points show evolutionary lineages proposed by Jacobs and Downs (1994) but does not indicate the evolutionary rate along the lineage.

The *Karnimata* clade (blue circles and light blue squares in figures) consistently shows greater distance from *A. chinjiensis* than the “*Progonomys* clade” (red diamonds in figures) at any given age, meaning that tooth shapes of species in the *Karnimata* clade are more modified than those of the *Progonomys* clade except *Mus* sp. at 7.4 Ma. After two sympatric species appeared in the region at 11.2 Ma, *Karnimata* of the *Karnimata* clade and the “*Progonomys* clade” experienced different degrees of morphological change. By 10.5 Ma, *Karnimata* sp. shows 60% morphological change, whereas *Progonomys* sp. (including specimens from 10.1 Ma) underwent 50% morphological change. The distance was constant at about 50% in *Progonomys* sp. by 8.2 Ma, whereas *Karnimata* sp. underwent over 75% change at 8.8 Ma and stayed at about 70% by 7.4 Ma. *Progonomys* sp. reached approximately the same degree of change as *Karnimata* sp. between 8.2 and 7.4 Ma, indicating delay in the modification of tooth shape in the “*Progonomys* clade” by at most 1.8 million years relative to *Karnimata*. At 7.4 and 6.5 Ma, *Karnimata* sp. and *Progonomys* sp. show similar degrees of morphological change. *Parapelomys*, which is considered to be derived from a species of *Karnimata* (Jacobs and Downs, 1994), shows greater amounts of shape change than *Karnimata* species, reaching 80% change in *Parapelomys* sp. at 7.4 Ma and 100% change in *P. robertsi*. *Parapelomys robertsi* has significantly more modified tooth shape than *Mus auctor* or *Karnimata huxleyi*.

### Patterns of tooth shape with ecomorphology and size

**Figure 8:**
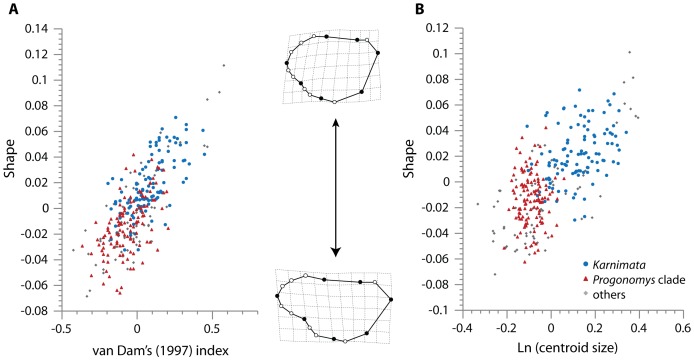
Scatterplots of the single pair of PLS axes (PLS1) from two-block partial least-square analyses. (A) van Dam's [54] index vs. Shape. (B) Natural logarithm of centroid size vs. Shape. See Dataset S1 for assignments of clades for individual samples.

In the PLS analysis, the first (single) pair of PLS axes (PLS1), which explains 100% of total covariation between the two blocks, shows a significant correlation (r = 0.76) between tooth shape and VD index (Figure 8, *p*<0.0001 in the permutation test). The RV coefficient is 0.39 (*p*<0.0001 in the permutation test). Along PLS1 of shape, individuals with greater VD index values possess a shortened anterior side of the tooth, by having a set of landmarks (9, 10, 1, 11, 12) located more posteriorly and a set of landmarks (2, 13, 14, 3) located more anteriorly, and a reduced posterior side of the tooth with a set of landmarks (4, 5, 16) located more anteriorly (Figure 8). These features correspond to the overall trend of the *Karnimata* clade. Individuals with smaller VD index values have an elongated anterior side and an unreduced posterior side of the tooth, which correspond to a typical shape of the *Progonomys* clade. When the two clades were analyzed separately in the PLS analysis, PLS1 indicated *Karnimata* has a stronger correlation (r = 0.73, *p*<0.0001) between the two variables than the “*Progonomys* clade” (r = 0.61, *p*<0.0001). Compared to the value of the RV coefficient for all taxa (RV = 0.39), the value is similar in *Karnimata* (RV = 0.35, *p*<0.0001) but reduced in the “*Progonomys* clade” (RV = 0.19, *p*<0.0001), indicating that tooth shape change in derived members of *Karnimata* is more tightly associated with VD index than shape change in derived members of the “*Progonomys* clade”.

In the multivariate regression analysis, the natural logarithm (Ln) of centroid size shows a significant correlation with tooth shape (*p*<0.0001) and predicts 13.5% of total shape variation. The result of PLS analysis also shows a strong correlation (r = 0.67) between tooth shape and Ln centroid size by the pair of PLS axes (Figure 8), rejecting the null hypothesis of complete independence between tooth size and shape in Siwalik murines (*p*<0.0001), and the RV coefficient is 0.31 (*p*<0.0001). Similar to the results with the VD index, individuals with larger centroid size have a rounded tooth shape as in derived taxa of the *Karnimata* clade, whereas individuals with smaller centroid size have an elongated tooth shape as in derived taxa of the “*Progonomys* clade” (Figure 8). Individuals of *Karnimata* are scattered along a line, whereas individuals in the “*Progonomys* clade” are more clustered (Figure 8). When the two clades are analyzed in the PLS analysis separately, *Karnimata* has a slightly stronger correlation (r = 0.46, *p* = 0.0003; RV  = 0.11, *p* = 0.0003) than the “*Progonomys* clade” (r = 0.34, *p* = 0.02; RV = 0.05, *p* = 0.04), implying that an allometric effect is stronger in *Karnimata* than in the “*Progonomys* clade”.

## Discussion

### Comparison with the hypothesis of Jacobs (1978) and Jacobs and Downs (1994)

**Figure 9:**
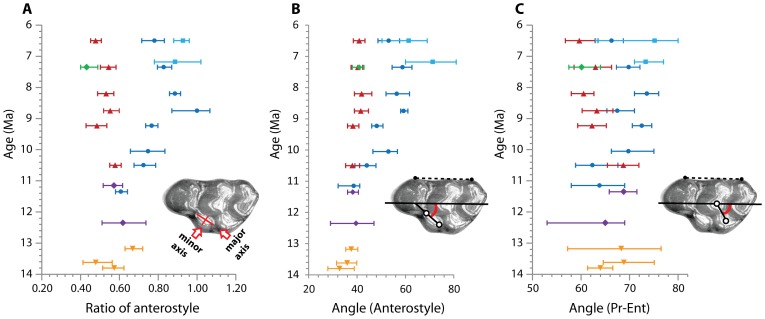
Temporal change of the three morphometric characters in Siwalik murines. (A) Ratio of the anterostyle, measured by the length of the minor axis relative to that of the major axis. (B) Acute angle between the anterostyle and the longitudinal axis of the tooth. (C) Acute angle formed by a line connecting the centers of protocone and enterostyle, and the longitudinal axis of the tooth. Symbols as in Figure 2. Abbreviations: Pr, Protocone; Ent, Enterostyle. Error bars indicate 95% bootstrap confidence intervals. Note that axes of the wear surface of the anterostyle parallel to the microscope lens. The specimen is *Progonomys debruijni* (YGSP 7740) in wear stage IV.

The results of the geometric morphometric (GM) analysis of tooth shape are summarized as follows. On PC1 and PC2, the morphospace of Siwalik murines is nearly triangular, apices of which are occupied by the earliest species, *Antemus chinjiensis*, and the two youngest species, *Mus auctor* and *Parapelomys robertsi* (Figure 4). Generally, the Euclidean distance from *A. chinjiensis* increases in species that occur in geologically younger ages, and the distance between two coexisting species becomes greater in younger ages (Figures 5 and 6) as the *Progonomys* clade shifts closer to *M. auctor*, while the *Karnimata* clade shifts closer to *P. robertsi*. These results support the phylogenetic hypothesis proposed by Jacobs [26] and Jacobs and Downs [19] that Siwalik murine rodents form dichotomous lineages descended from *Antemus*, the *Progonomys* and *Karnimata* clades (Figure 1). According to Jacobs [26], the *Progonomys* clade is characterized by the anterostyle located posteriorly, whereas the *Karnimata* clade is characterized by the anterostyle placed more anteriorly. In the GM analysis, this character was captured by the location of landmarks 2, 13, 4, and 3. This set of the landmarks is placed posterolabially in the *Progonomys* clade relative to the *Karnimata* clade. By observation of Siwalik murines, not only the location of the anterostyle but also its shape and angle are different between the two clades. The anterostyle is oval in the *Progonomys* clade and rounded in the *Karnimata* clade (Figure 9A, Table S4). The acute angles of the anterostyle relative to the longitudinal axis of the tooth are greater in the *Karnimata* clade (Figure 9B, Table S4). These characters can clearly differentiate the two clades without exception. The angle and shape of the anterostyle are similar between ?*Kanimata* and *P. hussaini* at 11.2 Ma in the beginning of the speciation event but became significantly different by 9.2 Ma in the angle and by 10.5 Ma in the shape.

The character measured as the angle of the enterostyle (i.e., angle made by a line passing through the center of protocone and enterostyle against the longitudinal axis of the tooth) is not explicitly listed as a character to differentiate the two clades in Jacobs [26]. However, he repeatedly mentioned the character in the systematic description of Siwalik murine species. According to Jacobs [26], “the enterostyle is posterior to the protocone and paracone” in *Progonomys debruijni*, and “the enterostyle is at the same level as the paracone” in *Karnimata darwini*, which coexisted with *P. debruijni* at 9.2 Ma. We measured the character as the angle of the enterostyle (Figure 9C, Table S4). The *Karnimata* clade tends to have a greater enterostyle angle than the *Progonomys* clade. However, every species has wider variation in this character than in the shape of the anterostyle, especially for *Antemus chinjiensis* and “near *Progonomys*”. The shape of the anterostyle is a better character than the angle of the enterostyle for identifying the two clades. The result of the GM analysis and the three morphometric distances are concordant with the observation-based conclusion of Jacobs [26].

### Lineage-specific morphological responses to diets

In derived species of the *Karnimata* clade, the occlusal surface of the two anterocones and the anterostyle are aligned more transversely than in the *Progonomys* clade, as observed in greater angles of the anterostyle in the *Karnimata* clade (Figure 9B). The morphological difference between the two clades is observed in the GM analysis as landmarks (2, 13, 14) associated with the anterostyle shift anterolingually, and landmarks (8, 9, 10, 1, 11) associated with the two anterocones shift posteriorly in the *Karnimata* clade. The posterior shifting of landmarks (8, 9, 10, 1, 11) effectively narrowed the space between the lingual anterocone and protocone in the *Karnimata* clade, and thus increased VD index values. In fact, more derived species of *Karnimata*, appearing later in time, have significantly greater VD index values [15]. A transverse alignment of the three anterior cusps has a functional advantage for murine rodents. Because the chewing motion of extant and fossil murine rodents is horizontal in the anteroposterior direction (but may be somewhat oblique in *Antemus*) [22], [35], [57], [58], [59], transversely aligned cusps are perpendicular to the direction of the mastication force and can grind food particles more efficiently than obliquely aligned cusps. Thus, shape change toward derived species of the *Karnimata* clade is closely related to increased chewing efficiency, providing a functional advantage for tough food in the diet.

In the *Progonomys* clade, the set of landmarks (10, 1, 11) shifts in the opposite direction to the corresponding set of the *Karnimata* clade. The results of the PLS analysis show that the anterior shifting of (10, 1, 11) decreased VD index values (Figure 8) although the linear regression analysis resulted in a constant VD index in the “*Progonomys* clade” between 9.2 and 6.5 Ma [15]. The major axis of the anterostyle is oblique to the longitudinal axis of the tooth, which is the direction of the mastication. Therefore, from a functional point of view, shape change toward derived species of the *Progonomys* clade may not be related to high grinding efficiency. There should be a functional advantage of having an elongated anterior part of the tooth in the *Progonomys* clade because the amount of shape modification from *A. chinjiensis* is lower in *Progonomys* than in *Karnimata* until 8.2 Ma but increased to the same amount by 7.4 Ma, by which both clades experienced a significant amount of C_4_ plants in their diet. However, the advantage is undefined in this study.

The fact that tooth shape in *Karnimata* (larger species, Figure 2) is better adapted to tough diets than that of *Progonomys* (smaller species, Figure 2) is concordant with their dietary preferences inferred by carbon isotope analysis and is reasonable regarding ecophysiological differences expected for their body sizes. *Karnimata* consumed higher proportions of C_4_ grass than the “*Progonomys* clade” although the rates of temporal change in carbon isotope values are not statistically significant from 9.2 through 6.5 Ma [15]. Generally, herbivorous mammals consuming a large amount of grass have modified teeth with adaptive traits (e.g., high tooth crown, lophodont teeth) to resist high rates of tooth wear due to soil grit or tough tissues [14], [60], [61], [62], [63], [64], [65]. Increased VD index in the *Karnimata* clade is similar to the adaptation of lophodont teeth by narrowing the space between chevrons. The shape change toward derived species of *Karnimata* is influenced by allometry more strongly than the “*Progonomys* clade”. Because gut capacity increases linearly with body mass, but mass-specific metabolic rate exponentially decreases with increasing body mass, larger herbivores require less energy per unit weight and are capable of acquiring nutrients from low quality foods more than smaller ones due to longer retention time for digestion [66], [67]. In rodents, Justice and Smith [68] demonstrated body mass is positively related to fiber consumption in four species of *Neotoma*. Smaller species selectively avoided fibrous particles from the same type of food pellets more than larger species [68]. The results of carbon isotope analysis suggest that larger-sized *Karnimata* consumed more of lower quality foods (i.e., C_4_ grass) than the smaller-sized “*Progonomys* clade” [15]. Therefore, selection pressure leading to shape change was differentially greater in *Karnimata* than in the “*Progonomys* clade”, and the functional and ecophysiological advantages acquired by derived species of *Karnimata* facilitated exploitation of C_4_ grasses, which spread between 8.5 and 6.0 Ma [7].

### Sympatric speciation as a result of competition for resources

The results of the GM analysis demonstrate gradual morphological change in Siwalik murines, which was qualitatively observed by Jacobs [26], and capture a pattern of sympatric speciation from one species through two morphotypes (?*Karnimata* and *Prognomys hussaini*) to two distinct species (*Karnimata* sp. and *Progonomys* sp.) in a quantitative manner. As the mean shape of the two clades deviated from each other (Figure 6), shape variation of one species that is closer to tooth shape of the other species (i.e., overlap area) diminished (Figures 5 and 6), resulting in progressive divergence of tooth shape through time. Between 11.4 and 10.1 Ma, when two morphotypes diverged into separate species, these species still consumed isotopically unimodal resources, suggesting they did not partition their dietary niche in the time interval [15]. As described above, the isotopic evidence suggests that coexisting species of *Karnimata* and the “*Progonomys* clade” developed isotopic dietary niche partitioning by 9.2 Ma with more C_4_ grass in the diets of *Karnimata* [15]. Habitat isolation, if not complete, was probably linked with morphological adaptation to different diets because C_4_ grasses flourished in the floodplain, whereas C_3_ plants persisted in more moist areas of the fluvial settings, such as abandoned channels at least until 4.8 Ma in the region [69].

The theoretical model of Dieckmann and Doebeli [70] indicates that disruptive selection, which favors opposite extremes of a trait within a single population [71], can emerge during sympatric speciation when the width of a unimodal distribution of resources is greater than the range of food items consumed by individuals ([70]; [72] for review). Three models proposed for ecological causes of sympatric speciation are based on resource competition, sexual selection, and habitat-specific mutation as reviewed in Turelli et al. [72] and Rundle and Nosil, [73]. Given the pattern of shape divergence in comparison with carbon isotopes in *Karnimata* and the “*Progonomys* clade”, disruptive selection led by interspecific competition for food sources between the two clades is the most parsimonious cause of the sympatric speciation event giving rise to the two clades. Although the possibility of speciation based on competition with other small mammals, predation, and sexual selection cannot be eliminated entirely, interspecific competition between the two clades is plausible for another reason described below.

From 9.2 through 6.5 Ma, when the *Karnimata* and “*Progonomys*” clades differentiated in tooth size (Figure 2) and shape (Figures 5 and 6), *Karnimata* significantly modified tooth shape earlier (i.e., between 9.2 and 8.8 Ma) than did the “*Progonomys* clade”. The latter stayed constant until 8.2 Ma and then diverged between 8.2 and 7.4 Ma to achieve the same amount of shape change as *Karnimata* (Figure 7). According to the theoretical model of Alizon et al. [74], which explains long unidirectional patterns of morphological evolution in cryptic species of fossil plankton, an inferior competitor can evolve only after a superior competitor has adapted to new ecological parameters under resource competition. The key finding of their study is that morphological change of the inferior competitor is delayed compared to that of the superior competitor when the cryptic species occur in sympatry and share the same food sources. The patterns of shape deviation of *Karnimata* and the “*Progonomys* clade” relative to *A. chinjiensis* (Figure 7) follow the model of Alizon et al. [74]. We neither suggest that *Karnimata* is ecologically superior to *Progonomys* nor that the model developed for gradual morphological change of fossil foraminifera can be applied directly to rodents. It should be mentioned that there is a difference in our study from the model in that *Karnimata* sp. and *Progonomys* sp. already acquired isotopic dietary niche partitioning during the time interval [15] although some overlap of resource use is not denied. However, we suggest that the sympatric speciation event and patterns of shape deviation from the basal species in the two clades are both explained most parsimoniously by their ecological interactions and lineage-specific adaptations to dietary shift due to the C_3_ to C_4_ vegetation shift in the region.

## Conclusions

Kimura et al. [15] documented, using carbon isotopes in enamel of molars, that Siwalik murine rodents experienced a significant dietary shift between 7.8 and 7.4 Ma by incorporating more C_4_ plants into the diet during the C_3_ to C_4_ vegetation shift. Their diets were isotopically similar until 10.1 Ma. From 9.2 to 6.5 Ma, *Karnimata* consistently had more C_4_ plants in their diet than the “*Progonomys* clade”. *Karnimata* evolved greater VD index as opposed to a constant VD index in the “*Progonomys* clade”, reflecting the dietary preference between the two clades shown isotopically. Here, we further explored lineage-specific responses of dental morphology to the dietary shift led by the ecological change, utilizing geometric morphometric analysis on the M1 outline. The morphospace defined by PCA of the shape data is the first quantitative dataset of Siwalik murine rodents and supports the phylogenetic hypothesis of dichotomous lineages resulting from sympatric speciation proposed by Jacobs [26] and Jacobs and Downs [19]. Tooth shape change in the *Karnimata* clade increased chewing efficiency for tough diets by narrowing the space between chevrons and having a transverse alignment of the three anterior cusps (lingual and labial anterocones, anterostyle) perpendicular to the power stroke in murine rodents. The functional interpretation of the *Progonomys* clade is inconclusive in this study, which is limited to two-dimensional outline shape and morphometric distances. Shape change in *Karnimata* is associated with increasing tooth size (i.e., increasing body mass) more tightly than in the “*Progonomys* clade”. Larger body mass accommodates low-quality food items to achieve required energy [66], [67]. Our results indicate that *Karnimata* was better adapted to consume C_4_ grasses than the “*Progonomys* clade” in both functional and ecophysiological aspects. Since the rates of temporal change in the intake of C_4_ grasses are the same between the two clades [15], selection forcing change in tooth shape was differentially greater in *Karnimata* than in the “*Progonomys* clade”.

Our data also suggest that disruptive selection observed in the two clades was most plausibly explained by ecological interactions, namely interspecific competition, between the two. The morphological evolution that differentiated the two clades initiated when two morphotypes consumed the same type of resource as indicated by carbon isotopic evidence. These observations are not contradictory to the theoretical model of Dieckmann and Doebeli [70] for sympatric speciation in the presence of resource competition. Interspecific competition may have hampered the “*Progonomys* clade” from shape modification until *Karnimata* exceeded a threshold of morphological innovation.

Sympatric speciation has been increasingly accepted as a likely process for the origin of sister species based on empirical evidence and convincing theoretical models ([72], [75] for review). However, the completion of sympatric speciation is often difficult to observe over the duration of normal empirical studies. Other factors in the ecological causes of sympatric speciation have not been tested and are in fact difficult to observe in fossils, but nevertheless the findings of this study show that a combination of shape analysis with carbon isotope analysis in in-situ evolutionary lineages of fossil small mammals can provide powerful paleontological evidence of the ecological causes of sympatric speciation. As in Siwalik murine rodents, the European lineage of murine rodents, *Progonomys*-*Occitanomys*-*Stephanomys*, is considered to result from anagenetic changes in size and dental morphology [54], [76], and a strong association of tooth shape and size was detected [33]. Direct comparisons of the Siwalik with European murine rodents or with other small mammals in the Siwaliks would provide deeper insight into ecological roles played during sympatric speciation of paleontological species.

## Supporting Information

Figure S1 **Tooth terminology used in this study. (A) Upper first molar. (B) Lower first molar.**
(PDF)

Figure S2 **Examples of wear stages I to VI in *Karnimata darwini.*** Wear stages I to IV correspond to those of Lazzari et al. [35]. From wear stage I to VI, specimens are YGSP 7692 (reversed), YGSP 7737, YGSP 7746 (reversed), YGSP 7667 (reversed), YGSP 7673, and YGSP 7725 (reversed), respectively.
(PDF)

Table S1 **A result of PCA on the covariance matrix of the shape data.** The 95% bootstrap confidence intervals were computed with 1000 randomizations in PAST.
(PDF)

Table S2 **Overlap area and Euclidean distances, corresponding to Figure 6.** CI for 95% bootstrap confidence intervals.
(PDF)

Table S3 **Euclidean distances from *Antemus chinjiensis* at 13.8 Ma to each species, corresponding to Figure 7.**
(PDF)

Table S4 **Summary of the three morphometric distances in M1 of Siwalik murine rodents, corresponding to Figure 9.**
(DOCX)

Dataset S1 **A dataset of individuals used in this study, including dental measurements (mm), van Dam's [54] index, centroid size, the three morphometric distances on occlusal surface, and the name of clades assigned for two-block partial least-square analyses (PLS).** Note that the “*Progonomys*” clade excludes *Mus* sp. at 7.4 Ma from the *Progonomys* clade of Jacobs [26]. Asterisks indicate individuals of *Karnimata* sp. from 7.4 Ma which were grouped with *Karnimata* sp. at 8.2 Ma.
(TXT)
