# Supplementary figures and images for: Lineage-Specific Responses of Tooth Shape in Murine Rodents (Murinae, Rodentia) to Late Miocene Dietary Change in the Siwaliks of Pakistan
Source: PLoS One. 2013 Oct 14;8(10):e76070. doi: 10.1371/journal.pone.0076070 (PMC3796524; doi:10.1371/journal.pone.0076070)

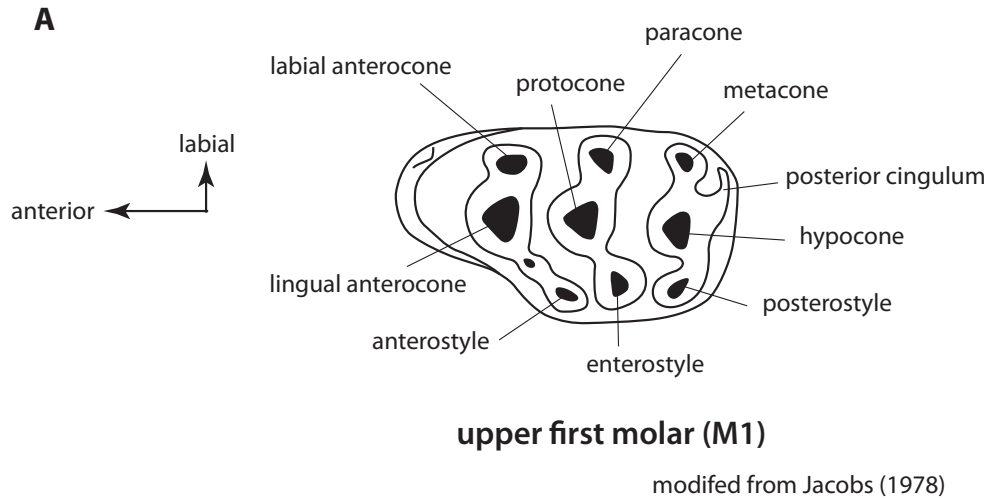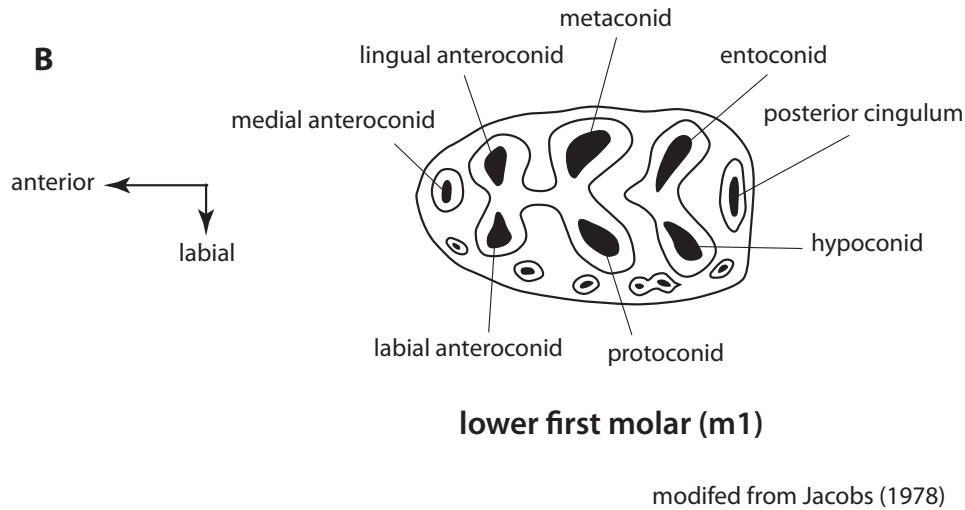

**Figure S1.** Tooth terminology used in this study. (A) Upper first molar. (B) Lower first molar.

Supplement: Figure S1 — Tooth terminology used in this study. (A) Upper first molar. (B) Lower first molar. (PDF) [file pone.0076070.s001.pdf]
