# Supplementary material for: Lineage-Specific Responses of Tooth Shape in Murine Rodents (Murinae, Rodentia) to Late Miocene Dietary Change in the Siwaliks of Pakistan
Source: PLoS One. 2013 Oct 14;8(10):e76070. doi: 10.1371/journal.pone.0076070 (PMC3796524; doi:10.1371/journal.pone.0076070)
Supplement: Figure S2 — Examples of wear stages I to VI in Karnimata darwini. Wear stages I to IV correspond to those of Lazzari et al. [35]. From wear stage I to VI, specimens are YGSP 7692 (reversed), YGSP 7737, YGSP 7746 (reversed), YGSP 7667 (reversed), YGSP 7673, and YGSP 7725 (reversed), respectively. (PDF) [file pone.0076070.s002.pdf]

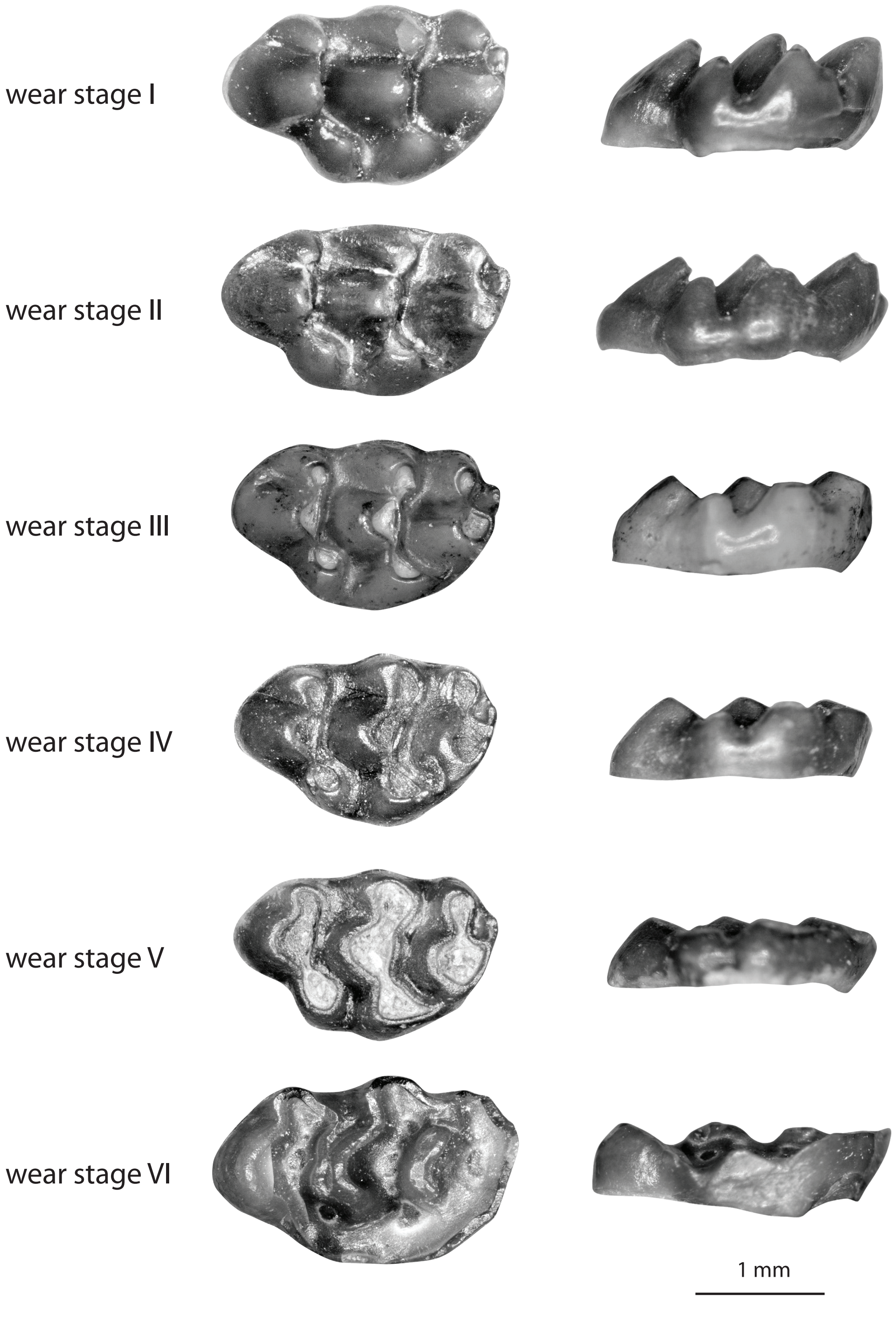

Figure S2. Examples of wear stages I to VI in *Karnimata darwini*. Wear stages I to IV correspond to those of Lazzari et al. (2008). From wear stage I to VI, specimens are YGSP 7692 (reversed), YGSP 7737, YGSP 7746 (reversed), YGSP 7667 (reversed), YGSP 7673, and YGSP 7725 (reversed), respectively.
