# Supplementary material for: Lineage-Specific Responses of Tooth Shape in Murine Rodents (Murinae, Rodentia) to Late Miocene Dietary Change in the Siwaliks of Pakistan
Source: PLoS One. 2013 Oct 14;8(10):e76070. doi: 10.1371/journal.pone.0076070 (PMC3796524; doi:10.1371/journal.pone.0076070)
Supplement: Table S1 — A result of PCA on the covariance matrix of the shape data. The 95% bootstrap confidence intervals were computed with 1000 randomizations in PAST. (PDF) [file pone.0076070.s003.pdf]

Table S1. A result of PCA on the covariance matrix of the shape data. The 95 % bootstrap confidence intervals were computed with 1000 randomizations in PAST.

Principal component	Eigenvalue	Variance (%)	95% bootstrap CI	
			Lower limit	Upper limit
1	0.000945	30.0	27.0	34.1
2	0.000720	22.8	19.9	25.7
3	0.000437	13.9	11.9	15.9
4	0.000218	6.9	5.7	8.2
5	0.000202	6.4	5.3	7.8
6	0.000144	4.6	3.7	5.3
7	0.000121	3.9	3.2	4.5
8	0.000088	2.8	2.2	3.2
9	0.000065	2.1	1.6	2.4
10	0.000055	1.7	1.4	2.0
11	0.000034	1.1	0.8	1.3
12	0.000030	1.0	0.7	1.1
13	0.000028	0.9	0.7	1.1
14	0.000016	0.5	0.4	0.6
15	0.000015	0.5	0.4	0.5
16	0.000012	0.4	0.3	0.5
17	0.000009	0.3	0.2	0.3
18	0.000006	0.2	0.1	0.2
19	0.000005	0.2	0.1	0.2
20	3.60E-07	0.0	0.0	0.0
21	2.47E-07	0.0	0.0	0.0
22	1.72E-07	0.0	0.0	0.0
23	9.15E-08	0.0	0.0	0.0
24	7.28E-08	0.0	0.0	0.0
25	4.56E-08	0.0	0.0	0.0
26	2.77E-08	0.0	0.0	0.0
27	2.22E-08	0.0	0.0	0.0
28	1.51E-08	0.0	0.0	0.0
