# Supplementary material for: Lineage-Specific Responses of Tooth Shape in Murine Rodents (Murinae, Rodentia) to Late Miocene Dietary Change in the Siwaliks of Pakistan
Source: PLoS One. 2013 Oct 14;8(10):e76070. doi: 10.1371/journal.pone.0076070 (PMC3796524; doi:10.1371/journal.pone.0076070)
Supplement: Table S2 — Overlap area and Euclidean distances, corresponding to Figure 6. CI for 95% bootstrap confidence intervals. (PDF) [file pone.0076070.s004.pdf]

Table S2. Overlap area and Euclidean distances, corresponding to Figure 6. CI for 95% bootstrap confidence intervals.

Age	Coexisting species 1		Coexisting species 2		Overlap area (%)	Distance			Note
(Ma)	Name	N	Name	N		Mean	Lower limit of CI	Upper limit of CI	
6.5	*Karnimata huxleyi*	10	*Parapelomys robertsi*	5	13.2	0.041	0.026	0.055	
6.5	*Mus auctor*	13	*Karnimata huxleyi*	10	0.0	0.091	0.072	0.107	
6.5	*Mus auctor*	13	*Parapelomys robertsi*	5	0.0	0.115	0.086	0.141	
7.4	*Mus* sp.	9	*Progonomys* sp.	8	10.0	0.037	0.022	0.049	
7.4	*Mus* sp.	9	*Karnimata* sp.	13	0.0	0.091	0.076	0.105	
7.4	*Progonomys* sp.	8	*Karnimata* sp.	13	0.0	0.060	0.042	0.070	
8.2	*Progonomys* sp.	11	*Karnimata* sp.	17	0.0	0.053	0.041	0.060	
8.8	*Progonomys* sp.	14	*Karnimata* sp.	5	4.3	0.049	0.040	0.067	
9.2	*Progonomys debruijni*	18	*Karnimata darwini*	36	8.3	0.042	0.032	0.051	*K. darwini*: 9.4, 9.2, 9.0 Ma, *P.debruijni*: 9.2, 9.0 Ma
10.1	*Progonomys* sp.	15	*Karnimata* sp.	9	12.3	0.040	0.025	0.054	*Karnimata*: 10.1 Ma, *Progonomys*: 10.1 to 10.5 Ma
10.5	*Progonomys* sp.	15	*Karnimata* sp.	11	19.7	0.031	0.018	0.040	*Karnimata:* 10.5 and 10.2 Ma, *Progonomys*: 10.1 and 10.5 Ma
11.3	*Progonomys hussaini*	35	? *Karnimata*	6	49.3	0.025	0.015	0.032	*P. hussaini*: 11.6, 11.5, 11.4, 11.3, 11.2 Ma + *Progonomys* sp. (12.3 Ma), ? *Karnimata*: 11.2 Ma
