# Supplementary material for: Lineage-Specific Responses of Tooth Shape in Murine Rodents (Murinae, Rodentia) to Late Miocene Dietary Change in the Siwaliks of Pakistan
Source: PLoS One. 2013 Oct 14;8(10):e76070. doi: 10.1371/journal.pone.0076070 (PMC3796524; doi:10.1371/journal.pone.0076070)
Supplement: Table S3 — Euclidean distances from Antemus chinjiensis at 13.8 Ma to each species, corresponding to Figure 7. (PDF) [file pone.0076070.s005.pdf]

Table S3. Euclidean distances from *Antemus chinjiensis* at 13.8 Ma to each species, corresponding to Figure 7.

Age (Ma)	Species	N	Mean	Lower limit of CI	Upper limit of CI	Percentage (%)	Combined age
6.5	*Parapelomys robertsi*	5	0.113	0.095	0.134	100	
6.5	*Karnimata huxleyi*	10	0.088	0.071	0.098	78	
6.5	*Mus auctor*	13	0.081	0.067	0.093	71	
7.2	*Parapelomys* sp.	3	0.090	0.072	0.103	80	7.1, 7.2, 8.0 Ma
7.4	*Karnimata* sp.	13	0.080	0.067	0.088	71	
7.4	*Progonomys* sp.	8	0.075	0.064	0.084	66	
7.4	*Mus* sp.	9	0.087	0.073	0.099	77	
8.2	*Karnimata* sp.	17	0.078	0.066	0.086	69	
8.2	*Progonomys* sp.	11	0.058	0.047	0.066	51	
8.8	*Karnimata* sp.	5	0.090	0.079	0.107	79	
8.8	*Progonomys* sp.	14	0.059	0.049	0.068	52	
9.2	*Karnimata darwini*	36	0.064	0.056	0.071	57	9.4, 9.2, 9.0 Ma
9.2	*Progonomys debruijni*	18	0.059	0.047	0.070	52	9.2, 9.0 Ma
10.1	*Karnimata* sp.	9	0.059	0.046	0.065	52	
10.5	*Karnimata* sp.	11	0.068	0.059	0.077	60	10.5, 10.2 Ma
10.5	*Progonomys* sp.	15	0.053	0.041	0.063	47	10.5, 10.1 Ma
11.2	? *Karnimata*	6	0.046	0.032	0.059	41	
11.4	*Progonomys hussaini*	35	0.041	0.032	0.046	36	12.3, 11.6, 11.5, 11.4, 11.3, 11.2 Ma
12.4	near *Progonomys*	5	0.038	0.019	0.054	34	
13.0	*Antemus chinjiensis*	7	0.022	0.010	0.028	20	13.2, 13.1, 12.8 Ma
13.6	*Antemus chinjiensis*	14	0.011	0.005	0.011	10	13.7, 13.6 Ma
