# Supplementary material for: Lineage-Specific Responses of Tooth Shape in Murine Rodents (Murinae, Rodentia) to Late Miocene Dietary Change in the Siwaliks of Pakistan
Source: PLoS One. 2013 Oct 14;8(10):e76070. doi: 10.1371/journal.pone.0076070 (PMC3796524; doi:10.1371/journal.pone.0076070)
Supplement: Table S4 — Summary of the three morphometric distances in M1 of Siwalik murine rodents, corresponding to Figure 9. (DOCX) [file pone.0076070.s006.docx]

**Table S4.** Summary of the three morphometric distances in M1 of Siwalik murine rodents, corresponding to Figure 9.

| Age (Ma) | Species | N | Ratio of anterostyle | | | | Angle (Pr-Ent) | | | | Angle (Anterostyle) | | | | Combined age |
| --- | --- | --- | --- | --- | --- | --- | --- | --- | --- | --- | --- | --- | --- | --- | --- |
|  |  |  | Mean | SD | Min | Max | Mean | SD | Min | Max | Mean | SD | Min | Max |  |
| 6.5 | *Parapelomys robertsi* | 5 | 0.93 | 0.04 | 0.87 | 0.98 | 75 | 7.5 | 63 | 81 | 61 | 10.0 | 50 | 70 |  |
| 6.5 | *Karnimata huxleyi* | 10 | 0.78 | 0.09 | 0.60 | 0.90 | 66 | 4.2 | 60 | 72 | 53 | 7.0 | 43 | 62 |  |
| 6.5 | *Mus auctor* | 12 | 0.48 | 0.05 | 0.40 | 0.58 | 60 | 5.3 | 52 | 69 | 41 | 4.1 | 34 | 49 |  |
| 7.2 | *Parapelomys* sp. | 3 | 0.89 | 0.12 | 0.78 | 1.02 | 73 | 3.2 | 71 | 77 | 71 | 10.6 | 60 | 81 | 7.1, 7.2, 8.0 Ma |
| 7.4 | *Karnimata* sp. | 13 | 0.83 | 0.07 | 0.74 | 0.94 | 70 | 4.5 | 61 | 77 | 59 | 7.5 | 45 | 70 |  |
| 7.4 | *Progonomys* sp. | 8 | 0.55 | 0.06 | 0.45 | 0.63 | 63 | 5.4 | 53 | 70 | 40 | 5.0 | 34 | 46 |  |
| 7.4 | *Mus* sp. | 8 | 0.43 | 0.06 | 0.37 | 0.55 | 60 | 4.5 | 55 | 68 | 41 | 3.3 | 35 | 44 |  |
| 8.2 | *Karnimata* sp. | 17 | 0.89 | 0.06 | 0.80 | 0.98 | 74 | 5.2 | 63 | 81 | 56 | 10.2 | 40 | 73 |  |
| 8.2 | *Progonomys* sp. | 11 | 0.53 | 0.07 | 0.41 | 0.64 | 61 | 3.8 | 55 | 66 | 42 | 5.9 | 34 | 53 |  |
| 8.8 | *Karnimata* sp. | 4 | 1.00 | 0.10 | 0.87 | 1.10 | 68 | 2.6 | 65 | 71 | 59 | 1.5 | 58 | 61 |  |
| 8.8 | *Progonomys* sp. | 14 | 0.55 | 0.07 | 0.45 | 0.68 | 63 | 6.1 | 55 | 75 | 42 | 5.6 | 32 | 54 |  |
| 9.2 | *Karnimata darwini* | 34 | 0.77 | 0.09 | 0.56 | 0.95 | 73 | 6.1 | 59 | 87 | 48 | 7.0 | 40 | 66 | 9.4, 9.2, 9.0 Ma |
| 9.2 | *Progonomys debruijni* | 18 | 0.48 | 0.12 | 0.26 | 0.68 | 62 | 6.3 | 51 | 75 | 38 | 5.2 | 31 | 48 | 9.2, 9.0 Ma |
| 10.1 | *Karnimata* sp. | 9 | 0.75 | 0.14 | 0.53 | 0.97 | 70 | 6.3 | 61 | 83 | 53 | 7.1 | 40 | 62 |  |
| 10.5 | *Karnimata* sp. | 11 | 0.72 | 0.09 | 0.60 | 0.91 | 62 | 6.9 | 50 | 72 | 44 | 7.2 | 31 | 53 | 10.5, 10.2 Ma |
| 10.5 | *Progonomys* sp. | 15 | 0.58 | 0.06 | 0.47 | 0.69 | 69 | 6.4 | 57 | 82 | 38 | 5.7 | 29 | 48 | 10.5, 10.1 Ma |
| 11.2 | ? *Karnimata* | 6 | 0.57 | 0.06 | 0.50 | 0.64 | 64 | 6.5 | 56 | 73 | 39 | 4.6 | 30 | 43 |  |
| 11.4 | *Progonomys hussaini* | 35 | 0.61 | 0.09 | 0.46 | 0.83 | 69 | 8.6 | 51 | 83 | 38 | 6.7 | 23 | 54 | 12.3, 11.6, 11.5, 11.4, 11.3, 11.2 Ma |
| 12.4 | near *Progonomys* | 5 | 0.62 | 0.12 | 0.46 | 0.79 | 65 | 7.0 | 53 | 70 | 40 | 9.6 | 26 | 51 |  |
| 13.0 | *Antemus chinjiensis* | 7 | 0.67 | 0.06 | 0.60 | 0.75 | 68 | 12.4 | 48 | 84 | 38 | 3.3 | 33 | 43 | 13.2, 13.1, 12.8 Ma |
| 13.6 | *Antemus chinjiensis* | 14 | 0.48 | 0.14 | 0.31 | 0.74 | 69 | 9.8 | 54 | 93 | 36 | 7.9 | 22 | 50 | 13.7, 13.6 Ma |
| 13.8 | *Antemus chinjiensis* | 12 | 0.57 | 0.10 | 0.40 | 0.69 | 64 | 4.5 | 57 | 71 | 33 | 9.5 | 21 | 50 |  |
